# Synthetic biology and genome engineering in support of human, agricultural, and environmental security

**DOI:** 10.3389/fbioe.2026.1820360

**Published:** 2026-07-06

**Authors:** Carolyn Riley Chapman, Julie Trolle, Dominika Daria Wawrzyniak, Jef D. Boeke, Ran Brosh

**Affiliations:** 1 The Multi-Regional Clinical Trials Center of Brigham and Women’s Hospital and Harvard, Boston, MA, United States; 2 Department of Medicine, Brigham and Women’s Hospital, Boston, MA, United States; 3 Department of Medicine, Harvard Medical School, Boston, MA, United States; 4 Section of Medical Ethics, Department of Population Health, NYU Langone Health, New York, NY, United States; 5 Department of Genome Sciences, University of Washington, Seattle, WA, United States; 6 Seattle Hub for Synthetic Biology, Seattle, WA, United States; 7 Institute for Systems Genetics, NYU Langone Health, New York, NY, United States; 8 Department of Biomedical Engineering, NYU Tandon School of Engineering, New York, NY, United States; 9 Department of Biochemistry and Molecular Pathology, NYU Langone Health, New York, NY, United States; 10 Department of Pathology, NYU Langone Health, New York, NY, United States

**Keywords:** biomanufacturing, bioremediation, biosecurity, biosensing, biotherapeutics, genome engineering, nucleic acid synthesis, synthetic biology

## Abstract

The rapid pace of innovation in synthetic biology and genome engineering elicits a need to reevaluate systems of oversight to ensure that biosafety and biosecurity safeguards are keeping up. Accordingly, the regulation of nucleic acid synthesis, an enabling technology for synthetic biology and genome engineering, is a current focus of political debate in the United States. However, to develop appropriate governance that neither under- nor overregulates technological development, policy leaders must also appreciate how advances in synthetic biology and genome engineering are being employed in the interests of biosecurity to support human health, manufacturing, food security, ecosystems and the natural environment. Synthetic biology can support the development of new therapeutics and health technologies, alternative biomanufacturing methodologies, food and agricultural innovations, environmental biosensors for monitoring infectious and toxic agents, and interventions aimed at preserving and restoring our natural environment. This Perspective provides an overview of current and potential benefits of synthetic biology and genome engineering, aiming to balance the broader societal discussion of potential risks—particularly in this special issue of the journal—with potential social, economic, and environmental value to individuals and society at large.

## Introduction

Synthetic biology (SynBio) has grown and evolved rapidly since emerging over 20 years ago (Cameron et al., 2014), in part enabled by advances in synthetic nucleic acid technology (Hughes and Ellington, 2017). Although its scope is variously defined (NIBIB, 2025; NHGRI, 2019; Hutchison III et al., 2010; Kuiken, 2022), SynBio can be succinctly characterized as editing genetic material in living organisms to enable new functionality (Aminian-Dehkordi et al., 2023).

In 2022, the United States Congress mandated the National Security Commission on Emerging Biotechnology (NSCEB) to develop recommendations to ensure the country’s leadership position in biotechnology for national security and economic purposes, given potential impacts on defense, healthcare, agriculture, energy, and manufacturing (NSCEB, 2025). In 2025, the NSCEB released recommendations for strengthening national and international standards to abate biosafety and biosecurity risks, whether deliberate or accidental (NSCEB, 2025). While stressing that risks must be addressed, the report’s main recommendation was that the United States government should prioritize and invest in biotechnology because of its immense potential to benefit humanity—and its economic and political importance (NSCEB, 2025).

The rapid pace of innovation in nucleic acid synthesis, SynBio, and large-scale genome engineering raises important questions regarding risks. Experts assert a need to evaluate whether systems of oversight at institutional, regional, national, and international levels are appropriate, effective, and keeping pace with technological advances (NSCEB, 2025; Mackelprang et al., 2022; Mui et al., 2025). In response to these concerns, a May 2025 United States Executive Order directed agencies to review and update biosafety and biosecurity policies (The White House, 2025). The United States Congress is actively considering new legislation, such as the proposed Biosecurity Modernization and Innovation Act, which would enhance control over the content of DNA synthesis orders (Cotton, 2026).

To develop appropriate governance that addresses risks without restraining innovation, it is important to weigh biosecurity risks against biosecurity benefits, broadly construed as supporting human health, biomanufacturing, food security and safety, and preservation of ecosystems and the natural environment. In this Perspective, we provide a concise and accessible overview of current and potential benefits of SynBio and genome engineering, to counterbalance the discussion of SynBio biosecurity risks—the focus of this special issue.

## Human health

Current and potential applications of SynBio to improve human health include the development of genetically customized research tools, such as human cell and animal models to better understand human disease (Pinglay et al., 2026; Zhang et al., 2023; Moore et al., 2021; Li et al., 2020). SynBio has also opened up the possibility of creating new human diagnostics, vaccines, and therapeutics by engineering living cells; approaches include engineered bacteria, viruses, yeast, or human cells that produce effector molecules and/or enzymatic activity as well as those that perform specific cellular activities in response to environmental signals (Cable et al., 2021; Bashor et al., 2022; Ruchała et al., 2026). Engineered cells can execute a variety of effects in response to different environmental cues—whether physical (e.g., ultraviolet light, temperature), chemical, or biological—and the modular nature of synthetic circuits offers efficiency and flexibility (Lim, 2022; Cubillos-Ruiz et al., 2021).

### Human cell and gene therapies

Advances in SynBio have enabled the development of genetically modified human cell therapies, including FDA-approved autologous chimeric antigen receptor (CAR) T cell therapies for hematological malignancies and hematopoietic stem cell (HSC)-based therapies for genetic hemoglobinopathies and metabolic diseases (U.S. FDA, 2025; John and Czechowicz, 2025). Scientists are also utilizing SynBio to address various challenges associated with CAR T cell therapy for solid tumors (Bashor et al., 2022); opportunities include engineering or “armoring” CAR T cells to co-express chemokine receptors, cytokines and/or utilize combinatorial approaches to improve cell trafficking, persistence in the tumor microenvironment, and antitumor activity (Kwon and Chen, 2025; Uslu and June, 2025; Cable et al., 2021). In clinical trials, CAR T therapies have demonstrated promise for treating some solid tumor cancers as well as autoimmune disorders (Uslu and June 2025). In the specific case of breast cancer, CAR T cells have not yielded desired clinical outcomes, but new SynBio strategies include engineering multiple CARs, combination strategies, suicide systems, and the generation of allogeneic CAR T cells (Buono et al., 2025).

Researchers are also engineering other types of human cells for potential therapeutic use (Chua et al., 2023). For example, T cells expressing an engineered T cell receptor (TCR-T cells), CAR-engineered natural killer (NK) cells, and CAR macrophages are also in development (Baulu et al., 2023; Zhang et al., 2022). SynBio is also being leveraged to optimize Regulatory T (Treg) cells to either augment their function in suppressing immunological responses (for autoimmune, allergy, and other hyperinflammatory disorders, as well as transplantation) or to otherwise interfere with it (for cancer) (Wardell et al., 2025; Tuomela et al., 2023). Of note, genetic engineering has also been utilized to create gene therapies that are delivered directly to humans (*in vivo*) via viral vectors or lipid nanoparticles (Li and Samulski, 2020).

### Microbial cell-based therapies

SynBio approaches are also being employed to develop microbial cell-based therapies to prevent or treat human disease (Cubillos-Ruiz et al., 2021; Jin et al., 2025). Microorganisms used in human therapy must be safe and amenable to genetic manipulation; these include *Lactococcus lactis*, which is present in fermented dairy foods, *Escherichia coli*, a commensal organism populating the human gut, and *Saccharomyces cerevisiae*, the “baker’s yeast” (Cubillos-Ruiz et al., 2021; Ruchała et al., 2026). Alternatively, SynBio may involve attenuation and manipulation of pathogens to effect immunological responses, e.g., against cancer (Liu et al., 2025; Cubillos-Ruiz et al., 2021). Scientists are furthermore working to engineer microorganisms to respond to environmental signals detected in the human gastrointestinal tract or in the solid tumor microenvironment (Charbonneau, 2020). A living microbial approach has also been taken to reduce dysbiosis associated with antibiotic use (Cubillos-Ruiz et al., 2022). To date, only non-genetically engineered Live Biotherapeutic Products (LBPs) have received FDA approval (e.g., Rebyota™ and Vowst™), but genetically engineered LBPs are in varying stages of research and development (Synapse, 2025; Brennan, 2022). For example, one group has demonstrated the potential of engineered probiotic *E. coli* Nissle 1917 to release a small molecule and anti-tumorigenic proteins to diagnose and treat adenoma, respectively, in a mouse model (Gurbatri et al., 2024).

### Transplantation

Solid organ transplantation is an established and life-saving treatment choice for patients with end-stage organ disease; worldwide, over 170,000 organs were transplanted in 2024 (GODT, 2026). Yet the number of patients in need vastly exceeds the availability of human donor organs (Zhang et al., 2023). To address this gap, scientists are investigating the feasibility of xenotransplantation, sourcing organs from different species, particularly pigs, which have anatomical and physiological similarities with humans, a short gestation period, and rapid growth (Vadori and Cozzi, 2024). SynBio techniques are employed to genetically engineer the animals to increase immunological and physiological compatibility and reduce the risk of transmission of porcine endogenous retroviruses (Yue et al., 2021).

Successful preclinical porcine-nonhuman primate xenotransplantation experiments, enabled by genome engineering of the pigs (Galli, 2025), have facilitated xenotransplantation in humans in recent years (Vadori and Cozzi, 2024). Porcine hearts, kidneys, and livers from gene-edited pigs have been transplanted into both brain-dead and living humans (Griffith et al., 2022; Porrett et al., 2022; Montgomery et al., 2022; Moazami et al., 2023; Cooper et al., 2025; Tao et al., 2025; Montgomery et al., 2025; Zhang et al., 2025). Although xenotransplantation would ideally provide a permanent solution, it may also serve as a bridge for patients awaiting a human organ (Tao et al., 2025; Cooper et al., 2025).

### Diagnostics and sensors

SynBio techniques are also being employed to develop microorganism-based and cell-free genetic sensors to advance diagnostic technologies and enable access in low-resource settings (Ostrov et al., 2017; Jin et al., 2025; Joshi et al., 2024; Kellner et al., 2019). The output of these systems can be limited to reporting the presence or absence of a particular biomarker, such as small molecules, nucleic acids, or protein; but more complex responses are also possible (Joshi et al., 2024; O’Connor et al., 2025). For example, one group engineered naturally competent (i.e., capable of taking up extracellular DNA) *Acinetobacter baylyi* to detect tumor DNA in both *in vitro* and *in vivo* (mouse model) systems (Cooper et al., 2023). In response to an environmental signal, genetic sensors may also stimulate the expression of specific biomolecules, that may be used for the delivery of therapeutics or biocontainment (Joshi et al., 2024).

Another promising avenue is the integration of bioelectronics and SynBio technologies to create biohybrid devices; however, these innovations are still at the proof-of-concept stage (Rivnay et al., 2025). One group designed an ingestible micro-bio-electronic device, combining engineered probiotic sensor bacteria with ultra-low-power microelectronics, that enabled *in situ* detection of blood (heme), a biomarker for gastrointestinal bleeding, in mice (Mimee et al., 2018).

## Biomanufacturing and biofuel production

Commercial manufacturers are leveraging SynBio technology to develop proprietary methodologies for efficient, cost-effective, climate-resilient, and/or environmentally sustainable production of a variety of materials to address supply chain challenges (Cumbers, 2026; Pfleger and Takors, 2023; Liu A. P. et al., 2022). For example, Conagen, a Massachusetts-based company, engineers living cells to produce amino acids and derive compounds, lipoflavoids, phenolics, polysaccharides, and oligosaccharides for use in food and other industries; they are also using SynBio approaches to develop and produce human monoclonal antibodies for therapeutic use (Conagen, 2026). Colorifix, in England, identifies unique pigments produced by various organisms and genetically engineers microorganisms to make large-scale production feasible (Colorifix, 2025). AmSilk, in Germany, employs SynBio to engineer strong silk proteins used to produce biomaterials (AmSilk, 2026). Bacterially-produced cellulose is harnessed by engineers as a material for use in textiles by Modern Synthesis in the United Kingdom (Modern Synthesis, 2026). SynBio approaches are also being investigated for optimization of microorganism-facilitated biocementation, which promises to be a sustainable alternative that could alleviate the environmental impact of conventional cement production (Dorfan et al., 2023; Carter et al., 2023).

Another SynBio application is biofuel optimization. Although fossil fuel combustion is currently the major global energy source for transportation and electricity, there is an urgent need to develop more sustainable fuels, due to concerns that the resultant greenhouse gases contribute to climate change, and that fossil fuels are limited, non-renewable, and will be depleted over time (Pfleger and Takors, 2023; Nath, 2024; LabXChange, 2022). Biofuels are a promising alternative and sustainable energy source, but challenges include feedstock availability and cost, impacts on land use and security, policy and market conditions, and technical hurdles (Nath, 2024). Notwithstanding these issues, synthetic biologists are designing and constructing new biological systems to expand the feasibility and scope of biofuel production, increasing sustainability, efficiency, and cost-effectiveness (Pfleger and Takors, 2023; Nath, 2024; Liu Z. et al., 2022). To accomplish this, researchers aim to engineer plants and microbes to speed up or alter biomass production, and design microbes that are more efficient at converting biomass into fuel (LabXChange, 2022).

## Food safety and security

Global food security is at risk due to population growth, climate change, environmental pollution, and reductions in arable land (Shi et al., 2022; Chen et al., 2025). To address food safety and security challenges, synthetic biologists aspire to fortify the nutritional content of foods, increase the efficiency and/or environmental resilience of agricultural crops, and develop new food production pathways (Sargent et al., 2022).

Fortification examples include the production of various strains of golden rice, which contain higher levels of β-carotene, with one variation also increasing the levels of iron and zinc (Ye et al., 2000; Barnum et al., 2021). Using multiplex gene editing, another group achieved a six-fold improvement of iron content in rice (Masuda et al., 2012). To improve the photosynthetic, nitrogen-use, and water-use efficiency of rice, a large international consortium known as the C_4_ rice project aims to add up to 20 genes to C_3_ rice (Sargent et al., 2022; Ermakova et al., 2020; Ermakova et al., 2021; The C4 Rice Project, 2025). Another efficiency-focused SynBio project aims to optimize plant carbon fixation by engineering the enzyme Rubisco to improve carboxylation, the first step of photosynthesis (Kubis and Bar-Even, 2019).

SynBio scientists are also developing new technology for food and dietary component production using renewable biomass, such as microorganisms (Chen et al., 2025). The goal of precision fermentation is to genetically engineer metabolic pathways in microorganisms to produce food ingredients with desirable properties (e.g., color, flavor, sustainability) from readily available substrates and/or in an environmentally-friendly and inexpensive way that reduces our dependency on fossil fuels (Hilgendorf et al., 2024; Shi et al., 2022; Aminian-Dehkordi et al., 2023). For example, companies have genetically engineered yeast to be capable of producing the proteins whey, casein, and lactoferrin (Nay, 2021; Reiley, 2023; Watson, 2025). SynBio can also be used to develop microbes with metabolic pathways that can utilize nonfood feedstocks, such as methanol and carbon dioxide, to create proteins and biomass (Shi et al., 2022).

Cultivated meat (*i.e.*, laboratory-derived, cell-based, cultured meat) offers a number of societal benefits, in terms of environmental sustainability, animal welfare, and reduced risk of foodborne illness (Kirsch et al., 2023). SynBio approaches may be useful for overcoming challenges with scalability in the production of cultivated meat from mammalian cells or for nutritional enhancement; however, the use of genetically modified cell lines will face regulatory and public acceptance challenges (Riquelme-Guzman et al., 2024; Stout et al., 2020).

## Environmental and biodiversity protection

SynBio is also being employed to preserve and restore the natural environment and/or arable land. As mentioned above, synthetic biologists seek to curb negative environmental impacts of greenhouse gas emissions by creating genetically engineered plants that have more efficient and/or novel carbon-capturing photosynthetic pathways (Erb, 2024, Dutta et al., 2021).

Researchers are also employing SynBio approaches to create pollutant monitoring driven by genetically engineered microorganisms, plants, or even cell-free systems (Joshi et al., 2024; Castaneda- Méndez et al., 2025; Aminian-Dehkordi et al., 2023; Jung et al., 2020). Similarly, bacteria are being engineered to serve as biosensors for the detection of environmental DNA (O’Connor et al., 2025). Scientists have also demonstrated that yeast can be engineered to detect pathogens in various samples, including blood, urine, and soil, suggesting diverse applications in human diagnostics and environmental surveillance (Ostrov et al., 2017).

SynBio can also be leveraged to develop bioremediation applications based on genetically engineered microbes, insects, or plants (Ruchała et al., 2026; Joshi et al., 2024; Castaneda- Méndez et al., 2025; Aminian-Dehkordi et al., 2023). Leveraging knowledge from systems biology approaches, environmental scientists are using metabolic engineering to alter microbes to be capable of neutralizing one or more pollutants, including per- and poly-fluoroalkyl substances (PFAS) (Olewade et al., 2025; Marchetto et al., 2021; Dangi et al., 2019). Other applications involve engineering microbes for the production or degradation of plastics (Choi et al., 2023).

Another direction for SynBio is in environmental conservation, including the preservation or restoration of ecosystem diversity (Lean, 2024). There are three main uses of SynBio in conservation: a) to engineer species’ genomes to stabilize their populations and protect against extinction (discussed in Brodie et al., 2025), b) to use gene drives or other genetic mechanisms to eliminate problematic (*e.g*., disease-causing) species (*e.g.*, Weng et al., 2024), and c) to alter environmental ecosystems through SynBio-facilitated de-extinction of species (*e.g*., Turner et al., 2025; Lean, 2024).

## Discussion

To balance the discussion in this special issue on biosecurity, this Perspective provides an overview of potential beneficial applications of SynBio in human health, biomanufacturing and biofuel production, food safety and security, and environmental protection.

SynBio poses different types of risks, including but not limited to biosafety and biosecurity risks, depending on the specific application and whether it is legitimate (i.e., intended to benefit human health, agriculture, or the environment), such as those reviewed here, or illegitimate (i.e., intentionally designed to cause harm), which are not the focus of this article. As SynBio can be leveraged for maleficent purposes, it is considered a dual-use research area of concern (Trump et al., 2021).

Even well-intentioned, legitimate SynBio projects have the potential for unintended, undesirable outcomes: many are in early stages of development and face scientific, technical, regulatory, and public acceptance challenges. For example, although Golden Rice has elevated levels of β-carotene, storage conditions and cooking methods impact its stability (Bollinedi et al., 2019), and the project has faced a general lack of acceptance in many countries (Kettenburg et al., 2018). More concerningly, legitimate SynBio projects may unintentionally harm humans, animals, or the environment. In the case of genetically modified cells and therapies, manipulation of the genome may result in unintended changes that could cause adverse health effects (e.g., Bantele et al., 2025; de Haart et al., 2025).

A complex set of governance frameworks at local, state, national and international levels aims to prevent illegitimate SynBio applications and minimize risks of legitimate ones (e.g., United Nations, 1972; U.S. FDA, 2024; Sun et al., 2022). Regulatory oversight of legitimate SynBio is appropriately tailored to different applications due to the need for expert evaluation of potential benefits and risks. These frameworks should be—and are—evaluated on an ongoing basis to keep up as technology advances (Evans, S. W. et al., 2026; Ou and Gou, 2023; Trump et al., 2021; Hamilton et al., 2021; Cotton, 2026; Buchman and Kovack, 2025).

To enable beneficial SynBio innovation while minimizing biosafety and biosecurity risks, policymakers must strike the right balance (Patrick and Barton, 2024). Under-regulation of SynBio risks preventable harm, whether accidentally or deliberately caused (Bohua et al., 2023). Yet over-regulation impedes beneficial applications (Millett et al., 2020; Smyth, 2017). Although Golden Rice has limitations, experts affirm its potential to save lives; yet the project has faced significant regulatory hurdles that have thwarted implementation, in part due to the Cartagena Protocol on Biosafety’s emphasis on assessing risks, but not potential benefits (Palmer, 2025; Wu et al., 2021).

Legitimate SynBio scientists must understand and adhere to the policies and regulations that govern their work. In coordination with stakeholders, they have an ethical obligation to carefully appraise both the benefits and risks of their projects—and communicate them to regulators, policymakers, and the public (Stilgoe, Owen, and Mcnaghten, 2013). The Safety and Security Program for the International Genetically Engineered Machine competition (iGEM) provides a model for this approach (Millett et al., 2020). To find the right path forward for SynBio policy, we must attend not only to SynBio’s risks, but also to its vast potential social, economic, and environmental value.

## Data Availability

The original contributions presented in the article are included in the article, further inquiries can be directed to the corresponding author.
